# Comment on Kim et al. The Association between Coffee Consumption and Risk of Colorectal Cancer in a Korean Population. *Nutrients* 2021, *13*, 2753

**DOI:** 10.3390/nu13124514

**Published:** 2021-12-17

**Authors:** Ravi Dhawan, Yuchen Zhao, Edward Giovannucci, Stephanie Smith-Warner

**Affiliations:** 1 Department of Epidemiology, Harvard T.H. Chan School of Public Health, Boston, MA 02115, USA; ravidhawan@hsph.harvard.edu (R.D.); yuchenzhao@hsph.harvard.edu (Y.Z.); 2 Department of Nutrition, Harvard T.H. Chan School of Public Health, Boston, MA 02215, USA

We read with interest the recent publication in *Nutrients* by Kim et al. [1] on the association between coffee consumption and odds of developing colorectal cancer (CRC) in a Korean population after adjustment for consumption of coffee additives. The authors offered insights on the potential protective effects of coffee consumption on CRC risk, but the study had limitations regarding its study design and data analysis that deserve comment.

To the best of our knowledge, no prior cohort or case-control studies have assessed the protective effects of coffee consumption on CRC in a Korean population [2,3]. In this novel study by Kim et al., no appreciable association was observed in the analysis after adjusting for age, sex, education, occupation, smoking, alcohol, total energy intake, physical activity, BMI, and first-degree family history of CRC in every analysis among women and men combined, by sex, or by tumor site (Model 2). However, after further adjustment for coffee additives (Model 3), the odds ratio (OR) for the risk of developing CRC among those drinking ≥3 cups of coffee/day compared to non-coffee drinkers decreased approximately two- to three-fold. For instance, the observed OR for CRC diagnosis among ≥3 cups/day drinkers vs. non-drinkers changed from 0.71 in the unadjusted model (Model 1) to 0.68 in the adjusted model (Model 2) to 0.22 after further adjustment for coffee additive use (Model 3). These results suggest strong protective effects of coffee consumption on CRC risk; however, this inverse association only emerged after further adjustment for cream and sugar additives. The authors correctly identified coffee additives as potential confounders. For example, it is plausible that the consumption of sugary beverages, especially early in life, may be associated with increased CRC risk [4,5]. Moreover, studies suggest that over 80% of the total coffee market in Korea consists of instant coffee mixes that contain sugar and cream additives [6,7]. From Table 1 [1], coffee additives intake increased linearly with coffee intake. The authors stated there was not a multicollinearity issue in their analyses. We are interested in how this was assessed as we question whether the independent effects of coffee and additives in this population can be teased statistically by simultaneous modeling. We recommend comparing coffee drinkers with high additive intake and separately coffee drinkers with low additive intake with non-drinkers of coffee, which will avoid potential collinearity. The present analysis may be limited by few high coffee drinkers with low additives, but it may provide a realistic perspective of the data in attempting to separate their effects.

We commend the authors for recognizing the potential confounding by additives and encourage the further exploration of analytic methods to clarify the impact of coffee additives on the coffee and CRC association.

## Data Availability

Not applicable.
